# Supplementary material for: Survey mode and nonresponse bias: A meta-analysis based on the data from the international social survey programme waves 1996–2018 and the European social survey rounds 1 to 9
Source: PLoS One. 2023 Mar 16;18(3):e0283092. doi: 10.1371/journal.pone.0283092 (PMC10019664; doi:10.1371/journal.pone.0283092)
Supplement: Attachment — Submitted filename: Plos1_review.pdf [file pone.0283092.s003.pdf]

The author analyses nonresponse bias approached by the “internal criteria of representativeness”. The nonresponse bias indicator (NBI) is given by the difference between the surveys proportion of females in two-person households from 0.5. The author analysis the effect of mode on NBI controlling for the sampling frame, interaction of sampling frame with mode, year of fieldwork, survey experience in a country, and an indicator for ESS surveys.

The analysis includes two-level random intercept models and eight different models for the meta-analysis. Here, the definition of the third level is varied, and the models include and exclude RR6. The author finds significant mode effects.

The topic is highly relevant. But I have some concerns about the method that should be addressed in a revised version of the manuscript.

1. As NBI, the share of women in two-person households is considered, which according to the theory should be 50%. An expectation of 50% only makes sense if two-person households are exclusively heterosexual couples. This is not plausible because there are many living arrangements. The assumption that there are equal numbers of homosexual female and male couples is made here in a side note, but is essential. This needs to be emphasized more. It would also help to explain how the mode comparisons are affected if this assumption is not met.
2. Page 5: you state that Kohler argues that the internal criterion of representativeness is not affected by item nonresponse. This is surprising and I would like to see some explanation of the rationale in the manuscript.
3. The article explicitly considers only nonresponse and excludes other sources of error. However, measurement errors can be very relevant here and can never be excluded when it comes to survey data. And these can be related to the mode and bias the analysis. For example, Felderer, Kirchner, and Kreuter (2019) find measurement error even for socio-demographic characteristics. The surveys have all been different and questionnaire effects cannot be ruled out either. This should be discussed more in the limitations.
4. I’m not sure about the expression of the standard error of the NBI. Intuitively I would think that the SE is \sqrt{prop_fem*/1-prop_fem/n}. Why is it not? Please add a reference or derivation. I’m not sure where the SE is used in the manuscript anyway.
5. The ESS is only conducted face-to-face and only in Europe. To what extent does it help to add the ESS data here? ESS, mode and country are highly correlated. Is it not possible that the ESS variable picks up parts of the mode effect (directly and indirectly being correlated with country? I would like to see the analysis without controlling for ESS.
6. To understand the data better, I would like to see the face-to-face columns split by ESS and ISSP in table 1.
7. The values of NBI are somewhere between 0 and 0.5. How is this reflected in the model?

In my opinion, there are too many models presented. I would prefer presenting either the clustering of countries by the gdp or region, not both.

I am not clear what the random intercept two-level model looks like. Is it in columns 4 and 8 in the table? Why is it needed here? What is the added value of figures 2 and 3, which if I understand correctly are based on the model? Why is this presented if the meta-analysis is superior? Please either elaborate the added value or delete parts of the analysis.

The meta-models are surprisingly robust when RR6 is added. But this is itself significant. How can this be even though the variables in the model explain RR6?

Knowing whether gender is correctly represented in the group of two-person households is certainly helpful. But what does it teach us about the survey as a whole? It may be that two-person households are completely misrepresented or that other variables are affected by nonresponse bias. This should be discussed more in the limitations.

Minor

To argue with changes due to the covid pandemic in the abstract is misleading. The data were collected before the pandemic. Changes in fieldwork by Covid 19 prove the importance of the study, but should rather be part of the introduction than the abstract.

Page 2, line 28: implementation **of** the survey

Page 4, line 67: the abbreviation NB needs to be introduced

Page 13. Line 273: I find this confusing. Please clarify when to use fixed and random effect models and add a reference

ESS: on page 4 you state that it is fully face-to-fact while later you claim that it is either CAPI or CATI. Please clarify
