# Supplementary material for: Survey mode and nonresponse bias: A meta-analysis based on the data from the international social survey programme waves 1996–2018 and the European social survey rounds 1 to 9
Source: PLoS One. 2023 Mar 16;18(3):e0283092. doi: 10.1371/journal.pone.0283092 (PMC10019664; doi:10.1371/journal.pone.0283092)
Supplement: Attachment — Submitted filename: survey review PLOS.pdf [file pone.0283092.s005.pdf]

Overall, I think this work is fit for publication, thought it feels borderline. In general, the author makes good use of the data they have access to but the data has some serious shortcomings and the types of mode under study are legacy modes at best in many settings. The inability to control for survey data climate is a major shortcoming (and I felt that this could be attempted, but not at this late point).

But overall, I think it an important enough contribution to merit publication. It makes a clear and compelling case for shifting away from the expensive face to face mode which is still commonly used in many countries. The expense of survey has gotten so extreme that it is now crowding out other data collection approaches and is limiting the span of topics we can investigate. The paper somewhat less relevant to other contexts. For example, in the North American context most of the focus with survey mode has long since moved on to focusing on modes like web/text/phone to field etc. It might be helpful to dwell on implications for those contexts (if any) or at least note how we can benefit from your methods. The data available for this study were both impressive and unimpressive. There were many surveys included (no small effort to compile all of this data). But the lack of auxiliary data is a serious shortcoming. As far as I could tell, NB was being assessed on a very narrow range of variables. And we don't know the extent to which these variables correlate with survey outcomes. The author should note this shortcoming.

The statistical work (meta regressions) all sounded right to me but I am not an expert in those methods so I rest on the impressions of other reviewers on that front.

The author cites the now well established finding that survey nonresponse bias is not clearly associated with response rates. It is worth delving in more detail as to whether this finding holds up in your study (there were some mentions here are there but I don't remember a bottom line takeaway.

Though its not the focus of this paper, social desirability bias which is closely tied to face to face surveys is a serious issue as well. The author might also consider referencing other implications because mode choice can affect the likelihood of survey falsification for example.

Generally, I feel that this article needs to tighten up the writing. It is choppy and informal in places, with far too many acronyms

Line 37/38: why not mention country effects? They would seem very important. And the fact that mode correlates so much with country feels like a threat to validity.

NB analysis seems very partial here due to the lack of auxillary data and a parallel set of outcomes like we have in most of our studies but this isn't the authors fault. it is offset by the sheer number of studies to some extent.

Line 94: Not their fault but representativeness on demographics is also no guarantee of survey data quality. Really depends on the correlations between outcomes and demographics. Mention this issue.

120: I found the reference to face to face surveys as the gold standard to be odd given that it is seldom used (in my context) any more. Maybe reorient this to a broader audience than the community of researchers working on these specific surveys?

Mode section: might want to discuss falsification as it can be an important issue given interviewer incentives

In the mode section the topic of escalation (starting with cheap modes like web) and then only going to expensive modes like face to face among late responders should be mentioned more. This has been shown to be a cost effective approach.

LINE 160 THIS PAPER IS NOT A LIT REVIEW IT IS AN EMPIRICAL STUDY. MAYBE SAY "THIS SECTION"

Line 171 Dillman didn't "prove" but rather "found" or "showed"

Literature review should be unpacked more. Lots of metrics flying around. The transition from the literature review to your research questions felt rushed. Needs better transition and need to take stock of the literature before rushing to your RQs

Line 194: Transition is weak. This needs smoother writing. Should say something in 194 like "given these findings, therefore, this study.. You just laid out many studies that do the same thing. What are you adding? Should make that clear around 197

Data and methods section is where the alphabet soup of acronyms made this hard to read particularly for those of us who don't work with these surveys. And don't abuse acroymns (for example do we really need to acronym "nonresponse bias"

PAPI/CAPI REFERENCE IS CONFUSING. IS THAT IN PERSON, BY PHONE? MY UNDERSTANDING IS THAT THOSE ARE TECHNOLOGIES NOT MODES.

Good use of AAPOR response rates. Methods section felt careful. Good attention to statistical details.

Around line 230 I got the impression that much of your work on representatives was soley based on gender. Is that really the case? Clarify.

needs a good edit. e.g. two grammar errors on line 246.

Line 258: the distribution of surveys by region is concerning but I know you are trying to adjust for it. Surprised that none of these surveys done by phone given proliferation of mobile tech.

Line 284: IN MY EXPERIENCE, EFFECT SIZE HAS A MORE PRECISE DEFINITION IN (EXPRESSING UNITS AS STANDARD DEVIATION CHANGES). The definition offered seems weak.

Line 317: Reassuring data is open source. I didn't have time to replicate the analyses.

318. I am confused why RR would be a mediator or a moderator. Feels like an outcome of mode choice.

Line 348: sentence fragment.

Line 358: intercepts would seem to capture lots of factors, not just country survey climate

Methods section: statistical methods seemed careful particularly with regard to weights/clusters but I have not used this type of model before so another reviewer should sign off on the methods.

Line 399: stereotypes wrong word>'expectations' is the better word.

443: this might be the software but table 2 is cutoff. This table could be more nicely presented.

Discussion and conclusion: makes some important and good points that are supported by the analysis. Maybe please widen the implications to other survey data collection contexts as mentioned above.

478: what risks? Face to face comes with its own serious risks that I think you might be underplaying.

Line 488: are you overselling paradata? Personally I haven't found it very useful despite the buzz in the literature a few years ago.

Line 499 I wondered about reverse causality: perhaps soften the language.

Line 521. I agree with this takeaway. we shoudl do more studies like this given the costs involved. Also administrative data miss a lot.

OVERALL, THIS FEELS LIKE AN IMPORTANT CONTRIBUTION PARTICULARLY IN COUNTRIES WITH STILL DO A LOT OF FACE-TO-FACE SURVEY DATA COLLECTION. There ARE IMPORTANT DATA LIMITIATIONS AND i DO FEEL LIKE ONE COULD COME UP WITH INSTRUMENTS FOR SURVEY CLIMATE BUT THAT FEELS LIKE A RESEARCH PROGRAM IN AND OF ITSELF. I HAVE SOME IMPORTANT EDITORIAL CONSIDERATIONS NOTED THROUGHOUT BUT THIS SEEMS WORTHY OF PUBLICATIONS. AS A DISCLAIMER I HAVE NOT USED METAREGRESSION SO I RELY ON THE OPINION OF OTHER REVIEWERS AS TO THE IMPLEMENTION OF THE SPECIFIC STATISTICAL METHODS USED HERE.

Note that my experience is specific to conducting surveys in the context of large randomized experiments in cases in which we have full frame data (including auxiliary data). Some of my comments might reflect my lack of familiarity with national survey efforts and if any of my comments are due to my lack of experience I apologize. The author did a good job given the data available and it feels important to save resources by moving away from face to face survey methods. I expect that there will be some additional benefits to moving away from face to face (or only using it as an escalation method for late responders.
