# Supplementary material for: Survey mode and nonresponse bias: A meta-analysis based on the data from the international social survey programme waves 1996–2018 and the European social survey rounds 1 to 9
Source: PLoS One. 2023 Mar 16;18(3):e0283092. doi: 10.1371/journal.pone.0283092 (PMC10019664; doi:10.1371/journal.pone.0283092)
Supplement: Attachment — Submitted filename: Responses to reviewers.docx [file pone.0283092.s004.docx]

**Responses to Reviewers of the manuscript titled "Survey mode and nonresponse bias: a meta-analysis based on the data from ISSP 1996–2018 and ESS rounds 1 to 9"**

**To all Reviewers:**

While considering the statistical suggestions I received in the revisions, I found some errors in standard error values in the database. I have corrected them and repeated every statistical procedure. It resulted in slight changes in the models but has not substantially changed the results.

I used your suggestion also to change the model specification and their number – currently, only three are presented in the body of manuscript, and two are in supplementary materials.

I used the opportunity of creating a new, corrected database and R script to upload them to the external repository instead of supplementary materials.

**Response to Reviewer 1:**

1. Follow-up analysis, passive and active nonresponse analysis and interest-level analysis can be effectively done only by the institution that has conducted or coordinated the survey because access to non-anonymized data about sample members is needed. The inclusion of such procedures usually is not reported in the survey methods reports.

2. Wave analysis is not applicable to the analysis of a large body of surveys from comparative programs with fieldwork organization hugely differing between countries. The fieldwork period is not identical even between ESS countries in each wave, which creates a problem with creating a standardized metric, and it is even more problematic with comparing early and late respondents between different survey modes.

3. Full replication of the whole survey of the country's population is usually not considered a viable solution to nonresponse bias assessment, because such endeavor’s financial cost is hard to justify.

4. Benchmarking is not applicable to "substantive" variables in analyses similar to those presented in this manuscript because topics covered in questionnaires differ between waves and programs. Therefore, multiple sources of differing quality and credibility are required as a benchmark.

Benchmarking based on demographic data comparison between one survey, and another depends on the decision, which of the two should be treated as a golden standard. Therefore, it has a very limited usage.

5. Comparison between the demographic composition of the net sample and official statistics is mentioned in the manuscript in the "nonresponse bias" section. Its main issue is that the quality of the official statistics may differ between countries as much as survey quality.

I added the reference (Halbesleben & Whitman, 2013) to the fragment describing the "external criteria of representativeness" – a comparison of demographic composition between respondents and the country's official statistics.

I added the following fragment at the end of the nonresponse bias section: "There are some additional possibilities for assessing the quality of the survey in terms of the nonresponse bias. One could use the gross sample information if it consists of individuals with known characteristics, but this is possible only in the case of individual samples (Johnson and Wislar, 2012). Additionally, nonrespondents could be targeted by very short follow-up questionnaires, as in ESS, but such follow-up may also be vulnerable to nonresponse. A similar vulnerability is shared by method comparing early and late respondents or individuals who needed the conversion procedures with the rest of the respondents (Halbesleben & Whitman, 2013). Such assessment methods are only possible for the secondary data analysis, when required information are easily accessible to external researchers, which is rarely the case."

**Response to Reviewer 2:**

1. I added these passages to address the issue of the inability to control for the same-sex couples: "For ESS rounds 1-8 and European Quality of Life Survey rounds 1-4, where controlling for same-sex couples is possible, the correlation between indicators using strict (with control) and lenient (as in this paper) approaches to asses NB was 0.96 and 0.93 respectively (Jabkowski, Cichocki, and Kołczyńska 2021). The inability to use a strict approach is an important limitation of this study."

2. I made the mistake of simply repeating Kohler's statement about criterion not being affected by item nonresponse, which obviously applied only to the surveys done with the participation of an interviewer. I added following sentence to address this: "(unfortunately only for face-to-face surveys, where gender is in most cases assessed by the interviewer)".

3. Focusing only on the nonresponse bias in my paper is a deliberate decision. I currently do not possess any data that make doing measurement error assessment at this scale possible. I have the NBI indicator based on the external UN demographic database in another dataset, but I find using it extremely problematic because of huge differences in official statistics quality between world regions, which is stressed in my manuscript.

Felderer, Kirchner, and Kreuter (2019) do not find measurement error for basic demographic characteristics, as used in my paper, only for those more prone to social desirability error, such as income or employment status. They write: "Not surprisingly, there is virtually no measurement error bias for gender in either mode (…) As expected, there is virtually no relative measurement error bias in mean age for either mode (…) there is no evidence for significant measurement error in any of the age categories, with relative measurement error biases being very close to zero". Additionally, they analyze data from only one country and with a linkage to administrative data for every respondent, which give them validation possibilities out of my reach.

As for the questionnaire (or topic) effects, in current models, they are at least partially accounted for by the random intercepts for waves, which shares the same (translated) questionnaires. Attitudes toward topics addressed in the questionnaires may differ between participating countries, but this is impossible to control on such a scale. Additionally, my paper is clearly focused on nonresponse bias. Total Survey Error paradigm made it possible to estimate different errors separately, and I am trying to do so for nonresponse.

4. I made the mistake of placing an explanation for the standard error calculation in a different part of the manuscript. To correct it, I repeated it just after the equation: "The standard error of this effect size is calculated based on the "true" gender proportion of 0.5". That is why prop_fem*(1-prop_fem)=0.5*(1-0.5)=0.5*0.5=0.25, as Kohler does it in the original paper. The inclusion of the equation is important, because it is clearly not obvious.

5. Inclusion of the "survey program" variable is important, because ESS and ISSP differ in much more areas than the used mode. I added references to papers describing these differences. I attach models without ESS data below to address the possibility of me choosing observations deliberately (I hope the output structure is not affected by the submission system). Code for models based on this subset of data is included in the R script for this paper, for readers information.

The model with "repetitions" variable included, ISSP only:

```
## Mixed-Effects Model (k = 572; tau^2 estimator: REML)
##
##     logLik    deviance         AIC         BIC        AICc
##  1143.7383  -2287.4766  -2267.4766  -2224.1438  -2267.0780
##
## tau^2 (estimated amount of residual heterogeneity):     0.0002 (SE = 0.0001)
## tau (square root of estimated tau^2 value):             0.0126
## I^2 (residual heterogeneity / unaccounted variability): 17.40%
## H^2 (unaccounted variability / sampling variability):   1.21
## R^2 (amount of heterogeneity accounted for):            67.71%
##
## Test for Residual Heterogeneity:
## QE(df = 563) = 656.0606, p-val = 0.0040
##
## Test of Moderators (coefficients 2:9):
## F(df1 = 8, df2 = 563) = 26.9885, p-val < .0001
##
## Model Results:
##
##                                                  estimate      se     tval   df    pval    ci.lb    ci.ub
## intrcpt                                            0.0437  0.0048   9.1516  563  <.0001   0.0343   0.0530  ***
## modMixed-mode (with f2f)                          -0.0058  0.0054  -1.0858  563  0.2780  -0.0164   0.0047
## modMail mode                                      -0.0116  0.0049  -2.3640  563  0.0184  -0.0212  -0.0020    *
## modMixed-mode (no f2f)                            -0.0127  0.0053  -2.3976  563  0.0168  -0.0230  -0.0023    *
## fraNonindividual frame                             0.0076  0.0044   1.7314  563  0.0839  -0.0010   0.0162    .
## rep                                               -0.0006  0.0002  -3.2584  563  0.0012  -0.0010  -0.0002   **
## modMixed-mode (with f2f):fraNonindividual frame   -0.0016  0.0074  -0.2115  563  0.8326  -0.0161   0.0130
## modMail mode:fraNonindividual frame                0.0663  0.0072   9.1598  563  <.0001   0.0521   0.0806  ***
## modMixed-mode (no f2f):fraNonindividual frame      0.0082  0.0137   0.6022  563  0.5473  -0.0186   0.0351

```

Model without "repetitions" variable included, ISSP only:

```
## Mixed-Effects Model (k = 572; tau^2 estimator: REML)
##
##     logLik    deviance         AIC         BIC        AICc
##  1141.2511  -2282.5022  -2264.5022  -2225.4867  -2264.1773
##
## tau^2 (estimated amount of residual heterogeneity):     0.0002 (SE = 0.0001)
## tau (square root of estimated tau^2 value):             0.0131
## I^2 (residual heterogeneity / unaccounted variability): 18.54%
## H^2 (unaccounted variability / sampling variability):   1.23
## R^2 (amount of heterogeneity accounted for):            65.12%
##
## Test for Residual Heterogeneity:
## QE(df = 564) = 668.0152, p-val = 0.0016
##
## Test of Moderators (coefficients 2:8):
## F(df1 = 7, df2 = 564) = 28.7200, p-val < .0001
##
## Model Results:
##
##                                                  estimate      se     tval   df    pval    ci.lb    ci.ub
## intrcpt                                            0.0343  0.0038   8.9528  564  <.0001   0.0268   0.0418  ***
## modMixed-mode (with f2f)                          -0.0063  0.0054  -1.1592  564  0.2469  -0.0170   0.0044
## modMail mode                                      -0.0099  0.0049  -2.0258  564  0.0433  -0.0196  -0.0003    *
## modMixed-mode (no f2f)                            -0.0122  0.0053  -2.2836  564  0.0228  -0.0226  -0.0017    *
## fraNonindividual frame                             0.0094  0.0044   2.1452  564  0.0324   0.0008   0.0180    *
## modMixed-mode (with f2f):fraNonindividual frame   -0.0030  0.0075  -0.4003  564  0.6891  -0.0176   0.0117
## modMail mode:fraNonindividual frame                0.0642  0.0073   8.8114  564  <.0001   0.0499   0.0785  ***
## modMixed-mode (no f2f):fraNonindividual frame      0.0089  0.0139   0.6409  564  0.5218  -0.0183   0.0361

```

6. I added the ESS survey numbers separately in the table.

7. Values of NBI are between 0 to 0.29, and model coefficients correspond with these values. Because of your suggestions, I decided to redesign the models. Currently, there are only 3 cross-classified models, with surveys nested simultaneously in countries and waves, with three different sets of moderators (simple, extended, and with partial effects). Additionally, I included two models: a two-level meta-regression model using NBI as an effect size and a three-level meta-regression model using RR6 as an effect size in the supplementary materials, for readers that do not wish to use supplemented R script.

8. While for one or a couple of studies, there are sometimes possibilities of using much more sophisticated assessments of unit nonresponse bias, for a couple hundred of surveys internal criterion is the most useful. Apart of the many papers I cited in the manuscript, that use this measure, also (Koch et al. 2014) used it to assess ESS sample quality: "This internal criterion for sample quality has the advantage that the same fixed benchmark (50%) can be applied to all countries. Furthermore, problems as regards the comparability of measurement do not compromise the analyses. On the downside, we have to restrict the analyses to one survey estimate (gender) and to a subgroup of the sample (respondents from gender heterogeneous couples".

Additionally, I added following sentences for clarification: "Additionally, it must be stressed that, in reality, nonresponse bias is a property of a specific variable. It can have different sizes for different survey questions. Because of that, the measure used here must be treated only as a proxy."

9. While I treated the abstract as a place to advertise the usefulness of a paper for possible reader, and that made me include pandemic consequences in it, following your suggestion I changed the sentence to: " Together with other unpredictable events occurring in the world today, this poses a challenge: the necessity to accelerate a switch from face-to-face data collection to different modes, which were usually considered to result in lower response rates. "

10. As far as I could find, I have written that ESS uses PAPI and CAPI, not CATI.

**Response to reviewer 3:**

1. The Previous manuscript included two mentions of limitations coming from the observational character of the data. I included two more, to stress this fact adequately.

A meta-analysis is a statistical analysis of a large collection of results from primary studies to integrate their findings (Glass 1976). While meta-analysis origins come from analyzing experimental data, it uses other types of data, even in the area of medicine and health studies, e.g. for disease frequencies estimation, or contraceptive prevalence estimation (Barendregt et al. 2013; Haakenstad et al. 2022; Kyu et al. 2022; Vos et al. 2020). In the survey methodology field, using observational data for meta-analysis is much more frequent. A paper analyzing the usage of this method in the field reports 28 manuscripts using meta-analysis on nonexperimental data from 54 included, more than a half (Cehovin, Bosnjak, and Lozar Manfreda 2018).

2. I included models based on different subsets of data in the R script for this manuscript, for readers to check if the results hold. I attach models based on ISSP only, and European-only data below (I hope the output structure is not affected by the submission system). The direction of results is very similar to one based on the full set. I used many possible methods to make this analysis as robust as possible. I specify them in the completely rewritten methods and results sections.

Model based on ISSP data only:

```
## Mixed-Effects Model (k = 572; tau^2 estimator: REML)
##
##     logLik    deviance         AIC         BIC        AICc
##  1141.2511  -2282.5022  -2264.5022  -2225.4867  -2264.1773
##
## tau^2 (estimated amount of residual heterogeneity):     0.0002 (SE = 0.0001)
## tau (square root of estimated tau^2 value):             0.0131
## I^2 (residual heterogeneity / unaccounted variability): 18.54%
## H^2 (unaccounted variability / sampling variability):   1.23
## R^2 (amount of heterogeneity accounted for):            65.12%
##
## Test for Residual Heterogeneity:
## QE(df = 564) = 668.0152, p-val = 0.0016
##
## Test of Moderators (coefficients 2:8):
## F(df1 = 7, df2 = 564) = 28.7200, p-val < .0001
##
## Model Results:
##
##                                                  estimate      se     tval   df    pval    ci.lb    ci.ub
## intrcpt                                            0.0343  0.0038   8.9528  564  <.0001   0.0268   0.0418  ***
## modMixed-mode (with f2f)                          -0.0063  0.0054  -1.1592  564  0.2469  -0.0170   0.0044
## modMail mode                                      -0.0099  0.0049  -2.0258  564  0.0433  -0.0196  -0.0003    *
## modMixed-mode (no f2f)                            -0.0122  0.0053  -2.2836  564  0.0228  -0.0226  -0.0017    *
## fraNonindividual frame                             0.0094  0.0044   2.1452  564  0.0324   0.0008   0.0180    *
## modMixed-mode (with f2f):fraNonindividual frame   -0.0030  0.0075  -0.4003  564  0.6891  -0.0176   0.0117
## modMail mode:fraNonindividual frame                0.0642  0.0073   8.8114  564  <.0001   0.0499   0.0785  ***
## modMixed-mode (no f2f):fraNonindividual frame      0.0089  0.0139   0.6409  564  0.5218  -0.0183   0.0361
```

Model based on European data only:

```
## Multivariate Meta-Analysis Model (k = 564; method: REML)
##
##     logLik    Deviance         AIC         BIC        AICc
##  1243.4970  -2486.9939  -2460.9939  -2404.8708  -2460.3198
##
## Variance Components:
##
##             estim    sqrt  nlvls  fixed  factor
## sigma^2.1  0.0000  0.0036     32     no    wave
## sigma^2.2  0.0000  0.0061     30     no   cntry
## sigma^2.3  0.0000  0.0016    564     no    surv
##
## Test for Residual Heterogeneity:
## QE(df = 554) = 575.4607, p-val = 0.2557
##
## Test of Moderators (coefficients 2:10):
## F(df1 = 9, df2 = 22) = 15.6551, p-val < .0001
##
## Model Results:
##
##                                                  estimate      se     tval   df    pval    ci.lb    ci.ub
## intrcpt                                            0.0480  0.0050   9.6354   20  <.0001   0.0376   0.0584  ***
## modMixed-mode (with f2f)                          -0.0080  0.0055  -1.4492  554  0.1479  -0.0189   0.0028
## modMail mode                                      -0.0180  0.0053  -3.4107  554  0.0007  -0.0284  -0.0077  ***
## modMixed-mode (no f2f)                            -0.0160  0.0049  -3.2632  554  0.0012  -0.0256  -0.0064   **
## fraNonindividual frame                             0.0004  0.0032   0.1313  554  0.8956  -0.0059   0.0068
## progESS                                           -0.0184  0.0042  -4.4053   22  0.0002  -0.0271  -0.0097  ***
## rep                                               -0.0008  0.0002  -3.3265  554  0.0009  -0.0012  -0.0003  ***
## modMixed-mode (with f2f):fraNonindividual frame    0.0095  0.0078   1.2294  554  0.2194  -0.0057   0.0248
## modMail mode:fraNonindividual frame                0.0690  0.0073   9.4339  554  <.0001   0.0546   0.0833  ***
## modMixed-mode (no f2f):fraNonindividual frame      0.0104  0.0118   0.8755  554  0.3817  -0.0129   0.0336

```

3. I used "sample quality" and "representativeness" to avoid repetitions. It is worth mentioning, that Kohler uses the term "internal criteria of representativeness" in his paper, and (Koch et al. 2014) gives the report, that uses Kohlers measure for ESS data the title: "Assessing ESS sample quality by using external and internal criteria". However, following your suggestion, I removed such terms from discussion section of manuscript, while leaving them in the introductory section, where they correspond to the claims of other authors.

4. Following your suggestion, I added the sentence: "Additionally, it must be stressed that, in reality, nonresponse bias is a property of a specific variable. It can have different sizes for different survey questions. Because of that, the measure used here must be treated only as a proxy." I summarize the conclusions of (Jabkowski et al. 2021), and give reference for this paper, which extensively analyzes the possibilities of measuring nonresponse bias for a large body of surveys conducted in different countries. Felderer, Kirchner, and Kreuter (2019), a paper suggested by another Reviewer, inquire into the subject of misreporting demographic data. For simple variables, not prone to social desirability bias (as used in the internal criterion) they state: "Not surprisingly, there is virtually no measurement error bias for gender in either mode (…) As expected, there is virtually no relative measurement error bias in mean age for either mode (…) there is no evidence for significant measurement error in any of the age categories, with relative measurement error biases being very close to zero".

5. I have rewritten the methods section to add more foundations to the choice of moderators and address their status in the possible causal inference model: "Choice of moderators for the models was based on both state-of-the-art and statistical evaluation. Survey mode and frame constitute probably the most basic set of survey characteristics. The subject of mode was extensively described in the introductory parts of this paper. As for the sampling frame, the frame of individuals is usually considered better in terms of representativeness than other types of frames, primarily because of the burden connected to the process of within-household-selection of the respondent at the last sampling stage (Gaziano 2005; Jabkowski 2018; Menold 2014). Therefore, I used the binary variable individual/nonindividual (household/address, area probability sampling). To not overcomplicate the model, I ignored the possible impact of the within-household selection method on NB (Jabkowski 2017). Exploratory subgroup analysis and previous knowledge about possible issues connected with the aforementioned respondent selection not being supervised by the interviewer (as in e.g. mail mode) led to the conclusion, that also the interaction between those two moderators may be an important factor in explaining differences in NBI values (Reader can conduct all analyses mentioned in the article, and some more, using the R script available at …). Therefore mode, frame, and interaction between them were selected for the simplest model specification. The analyzed dataset consists of studies from two survey programs, that differ significantly in terms of funding and organization, (Bréchon 2009; Smith and Fu 2016) hence the inclusion of binary variable ESS/ISSP in the extended model. Institutional experience can influence outcome rates of a survey, (Stoop et al. 2010:102–13) and thus at least the upper boundary of possible nonresponse bias. I chose to include a moderator, that for each observation simply counts how many surveys were done previously in this country and program. This study, as stated previously, is based on observational data – like many meta-analyses in survey methodology (Cehovin et al. 2018). If one, however, would like to apply the categories of variables usually included in the causal inference models, then this study is focused chiefly on the effect of survey mode on nonresponse bias, and every candidate for the meta-regression moderator listed above should be treated as a confounder. Sampling frame, institutional experience, and survey program can usually have an impact on the choice of mode(s) and separately on the nonresponse bias. As for the last candidate for a moderator – the response rate (RR6) – its inclusion can be used to estimate the partial effects of survey modes holding RR constant. In the causal inference model, it would be a mediator. RR6 was included only in the last of the models presented in the results."

6. While I am clearly not placing my reasoning in the causal inference paradigm, which would be extremely interesting, and following your suggestions, I am thinking about conducting different studies using some kind of matching technique, I can specify what would be the role of my moderators in my model. It is shortly mentioned in the modified parts of the manuscript cited above, but here I would like to elaborate further. The sampling frame chosen for the specific survey impacts both the choice of mode (e.g. some forms of area probability sampling are not possible to conduct without at least face-to-face screening, a necessity to conduct within-household selection will discourage use of modes, where there is no interviewer to at least supervise the selection, etc.) and the nonresponse (burden of within household selection resulting in more refusals, possibility of using fully personalized communication while having access to individual register, etc.). The survey program similarly impacts the choice of modes (as ESS does not give any choice) and the nonresponse (e.g. because of substantial funding differences). Finally, "repetitions" will also impact both the choice of modes (previous experience may lead to innovations) and the nonresponse (a more experienced team will implement the chosen survey design better, at least usually). I have excluded the year variable from the models, both because it lacked strong theoretical foundations, and because of statistical evaluation.
