# Supplementary material for: Survey mode and nonresponse bias: A meta-analysis based on the data from the international social survey programme waves 1996–2018 and the European social survey rounds 1 to 9
Source: PLoS One. 2023 Mar 16;18(3):e0283092. doi: 10.1371/journal.pone.0283092 (PMC10019664; doi:10.1371/journal.pone.0283092)
Supplement: Attachment — Submitted filename: Response to reviewers.docx [file pone.0283092.s006.docx]

I would like to thank the reviewers very much for suggesting many important improvements, which I have tried to implement in my manuscript. Additionally, below I present my responses to some issues.

**To Reviewer #2**

1. I have changed the part of my paper where I describe the chosen effect size to:

“NBI, being in this context a single study effect size, was calculated as:

ES_i_ = |prop_fem_ - 0.5|

where prop_fem_ is the proportion of females in a subsample of couples living together in two-person households and n is the size of a subsample of couples living together in two-person households (Kohler 2007). As the proportion on which the ES_i_ is based is only an estimate and the true proportion of females in the defined subpopulation is already known to equal 0.5, the within-study variance may be calculated as:

V_i_ = 0.25/n_i_

Where n_i_ is the subsample size of the single study (Kohler 2007; Jabkowski and Cichocki 2019; Eckman and Koch 2019).”

If I used the proportion in the survey subsample as a numerator in the variance equation, the calculation would assign very low variance to the subsamples with a very high or low proportion of females (sometimes even 1 or 0). This approach does not make sense because this “wrong” proportion is often caused by the tiny size of the subsample, resulting from some traditional societies not having many two-person households of partners. It would assign these studies much higher weight. If we use the known proportion of 0.5, these studies have a lower weight, as intended.

If we use the equation for the variance of the difference between proportions V = (p1 * (1 - p1) / n1) + (p2 * (1 - p2) / n2), n1 will be the size of the country’s population “living together in…,” estimated by multiplying the country’s population above a certain age for a given year and proportion in the survey subsample. This would put numbers like a couple of millions in the denominator, making the first part of the equation nearly irrelevant. The second part of the equation would be the same as I described in the previous paragraph, with the same problems. Using Vi = 0.25/n, like the cited authors, is the best option.

2. Looking at the forest plots, I do not conclude that there is a connection between RR6 and bias. I have slightly rewritten this paragraph to make it even clearer: “As we can see in figure 2, simple random-effects meta-analysis in subgroups shows that NBI values for mixed-mode surveys with face-to-face (mostly face-to-face with additional self-administered questionnaires) and for the mail mode clearly overlap with the estimate for single-mode face-to-face surveys. At this level, it is not possible to tell whether values of bias estimate for other mixed-mode surveys are smaller than for face-to-face. For context, we can see in figure 3 that the values of RR6 for modes using face-to-face surveys in any configuration clearly outperform other ones, which is in accordance with common expectations concerning this subject. Based on the presented literature, it was expected that the connection between realization and bias would be weak at most.”
3. It is true that I have treated the interpretation of insignificant interactions too lightly. I have rewritten parts of the Results and Discussion section to make it clearer that in this configuration of moderators you can only compare one frame type at a time, and I added a comparison model with the reference level of frame reverted mentioned in the text, and in the script.
4. Table 2 appears in such way, because the manuscript is converted to a PDF format. I think I sticks to the formatting guidelines; if not, I will discuss this topic with the editor. For the reviewers, I previously attached table 2 as a separate file and it is still available as an attachment.

**To Reviewer #4**

1. I changed all the NBs back to “nonresponse bias.” Therefore, now the only acronym which is not conventional in the field (along with RR, ESS, ISSP) is NBI (nonresponse bias indicator), which I am really convinced should stay in this abbreviated form to avoid constant repetition of such a long term.
2. I must admit that the quick step from the summary of the state of the art to the research questions was my intention, connected to the intention of creating a certain feeling in the reader (that the findings are confusing, that there are a lot of gaps in the knowledge, and that therefore this study is needed). However, following your advice, I expanded these two sentences into a full paragraph to make the transition smoother.
3. While local or commercial survey research has already mostly switched to web or phone designs, and in this area differences between PC and mobile are far more important than face-to-face vs. mail, these issues still seem essential in the big comparative projects focused on statistical representativeness. Not even the COVID-19 pandemic has forced behemoths like ESS or Eurobarometer to fully drop face-to-face. The only big program that switched to CATI in full in response to the restrictions was LAPOP (the AmericasBarometer), but it uses sophisticated quotas on household level and is therefore hard to consider methodologically driven. I added these sentences to the limitations section: “The last limitation worth mentioning here is the fact that this paper covers modes and approaches used mostly in large comparative survey programs, which are relatively well funded, based on international collaboration, and usually academically and/or methodologically focused. For researchers dealing with smaller surveys, usually local, or especially commercially focused, and who are not used to pursuing full statistical representativeness, the data presented here may be, unfortunately, of small importance. The author is fully aware of this limitation.”
4. I state the following in the nonresponse bias section: “Additionally, it must be stressed that nonresponse bias is a property of a specific variable. It can have different sizes for different survey questions. For this reason, the measure used here must be treated only as a proxy.” This whole section is devoted to explaining how hard it is to assess the bias, especially if no auxiliary data are present. I base my confidence in choosing the Kohler’s approach (even without controlling for same-sex couples and using weights) on the analysis by Jabkowski, Cichocki, and Kołczyńska (2021, doi: 10.1093/jssam/smab027) that focused clearly on the selection of bias indicators for this type of data and concluded that this method is optimal. The method, as I explain in my paper, is based on the proportion of genders in a defined subsample of two-person households of couples. As for the impact of different motivations and incentives for interviewers on the respondent selection process, Eckman and Koch (2019, doi:10.1093/poq/nfz012) used the Kohler measure to assess the bias coming from that source. Therefore, it may be considered covered in my analysis, because there is no motivation for interviewers to keep a certain proportion of genders in the specific subsample.
5. As for the “causal inference paradigm” terms (suggested by the previous reviewer), I have arbitrarily chosen to focus on the mode as a variable of main interest. In that situation, frame, program, and “repetitions” work clearly as confounders. The sampling frame chosen for the specific survey impacts both the choice of mode (e.g., some forms of area probability sampling are not possible to conduct without at least face-to-face screening; the necessity of conducting within-household selection will discourage the use of modes in which there is no interviewer to at least supervise the selection, etc.) and the nonresponse (once more, the burden of within-household selection, the possibility of using personalized communication while sticking to an individual frame, etc.). The survey program similarly impacts the choice of modes (as ESS does not give any choice) and the nonresponse (because of huge funding differences). Finally, “repetitions” will also impact both the choice of modes (previous experience may lead to innovations) and the nonresponse (A more experienced team will implement the chosen survey design better, at least usually.) RR6 is a mediator, as it is partially determined by the survey characteristics, and it also have **some** effect on nonresponse bias.
6. My data suggests that there is a significant relation between RR and NBI. The higher response rate, the lower the bias. It is visible in the model with the inclusion of RR6 as one of the moderators, and if one used it as a sole moderator in that model, the relationship would be even stronger. I have consciously omitted elaboration on this subject in my paper, because a reviewer from *Survey Research Methods* (where I sent the earliest version of this paper, containing the mentioned model) advised me that the question of pure relation between RR and bias was interesting 10 years ago and that I should focus on the subject of lowering the bias. The data and scripts are available and fully commented on by me. As I now see this subject not as important, I would prefer to leave it in the script, for the very interested researchers.
7. PAPI/CAPI are “pen & paper” and “interviewer with a laptop” variations of face-to-face. It does not make for a huge difference (which is why I do not account for it in my models), but for some researchers it is important, like the PC/mobile difference in CSAQ (while others even see PC and mobile as separate modes).
8. I am currently ending a project that maps survey designs in big, comparative, noncommercial survey projects around the globe, and the only one that used a large portion of phone interviewing was LAPOP. There is generally a problem with running statistically driven surveys by phone, because of issues with the frame (or especially because of the lack of it).
9. I have expanded the paragraph defining what an effect size is. It is mainly targeted to reduce possible confusion that readers used to standard measures used in clinical research and similar may feel. Usage of NBI as an effect size in meta-analysis was performed in a couple of papers that I cite. The paragraph now reads as follows: “Most commonly, meta-analysis is used to create proper statistical synthesis of results taken from many articles or clinical trials. In that situation, some typically used dependent variables, called effect sizes or treatment effects (Borenstein et al. 2009, 17), are usually computed to either statistically standardize the different measures used by different authors or comply with standard settings of experimental studies in medicine. Therefore, readers probably encountered two types of effect sizes: ‘those based on differences of two means and those based on differences of two proportions’ (Hartung, Knapp, and Sinha 2008, 13). However, these examples do not exhaust the usefulness of meta-analysis. On the contrary, “effect size is simply the size of anything that may be of interest” (Cumming 2012, 34), whether it is measured in exactly the same way for every study or recalculated to make it so. For example, in epidemiological studies, the true population mean, or proportion, may be of interest (Barendregt et al. 2013). Card proposed that the working definition of effect size could be “an index of the direction and magnitude of association between two variables” (Card 2012, 87), a definition that would include the measure used in this paper, if interpreted *sensu largo*.
10. As for the paradata, it is important in the studies on the European Social Survey methodology on one side, and on the other, it is an important part of the responsive/adaptive/tailored survey design that has been implemented with some success, at least in the Netherlands.
11. I also implemented many minor corrections suggested by the reviewer.
